# Phylogenetic Characterization of Novel Reassortant 2.3.4.4b H5N8 Highly Pathogenic Avian Influenza Viruses Isolated from Domestic Ducks in Egypt During the Winter Season 2021–2022

**DOI:** 10.3390/v16111655

**Published:** 2024-10-23

**Authors:** Noha Saad, Mana Esaki, Isshu Kojima, Ahmed Magdy Khalil, Shiori Osuga, Momtaz A. Shahein, Kosuke Okuya, Makoto Ozawa, Bader Y. Alhatlani

**Affiliations:** 1 Animal Health Research Institute, Agricultural Research Center, Giza 12618, Egypt; momtaz.shahein@yahoo.com; 2 National Laboratory for Veterinary Quality Control on Poultry Production, Animal Health Research Institute, Ministry of Agriculture, Giza 12618, Egypt; 3 Department of Pathogenetic and Preventive Veterinary Science, Joint Faculty of Veterinary Medicine, Kagoshima University, Kagoshima 890-0065, Japan; k0068103@kadai.jp (M.E.); i-kojima@ous.ac.jp (I.K.); ahmed.magdy.am549@gmail.com (A.M.K.); shiorin_birdie@yahoo.co.jp (S.O.); kokuya@vet.kagoshima-u.ac.jp (K.O.); mozawa@vet.kagoshima-u.ac.jp (M.O.); 4 Joint Graduate School of Veterinary Medicine, Kagoshima University, Kagoshima 890-0065, Japan; 5 Department of Zoonotic Diseases, Faculty of Veterinary Medicine, Zagazig University, Zagazig 44511, Egypt; 6 United Graduate School of Veterinary Science, Yamaguchi University, Yamaguchi 753-8515, Japan; 7 Transboundary Animal Diseases Research Center, Joint Faculty of Veterinary Medicine, Kagoshima University, Kagoshima 890-0065, Japan; 8 Unit of Scientific Research, Applied College, Qassim University, Buraydah 52571, Saudi Arabia

**Keywords:** avian influenza virus, ducks, H5N8, influenza, whole-genome sequencing, reassortment

## Abstract

Avian influenza (AI) is an extremely contagious viral disease of domestic and wild birds that can spread rapidly among bird populations, inducing serious economic losses in the poultry industry. During the winter season 2021–2022, we isolated seventeen highly pathogenic avian influenza (HPAI) H5N8 viruses from outbreaks involving ducks in Egypt, occurring in both backyard and farm settings. The aim of this study was to pinpoint genetic key substitutions (KSs) that could heighten the risk of a human pandemic by influencing the virus’s virulence, replication ability, host specificity, susceptibility to drugs, or transmissibility. To understand their evolution, origin, and potential risks for a human pandemic, whole-genome sequencing and phylogenetic analysis were conducted. Our analysis identified numerous distinctive mutations in the Egyptian H5N8 viruses, suggesting potential enhancements in virulence, resistance to antiviral drugs, and facilitation of transmission in mammals. In this study, at least five genotypes within one genome constellation of H5N8 viruses were identified, raising concerns about the potential emergence of novel viruses with altered characteristics through reassortment between different genotypes and distinct groups. These findings underscore the role of ducks in the virus’s evolutionary process and emphasize the urgent need for enhanced biosecurity measures in domestic duck farms to mitigate pandemic risk.

## 1. Introduction

Avian influenza (AI) is an extremely contagious viral disease of domestic and wild birds that can spread rapidly among bird populations, inducing serious economic losses in the poultry industry [1]. The disease is caused by avian influenza virus (IAV), which is a member of the influenza A viruses (IAVs) within the family *Orthomyxoviridae*. IAV is an enveloped, negative-sense, single-stranded, segmented RNA virus [2,3]. IAVs are categorized based on the surface proteins hemagglutinin (HA) and neuraminidase (NA) into 16 HA subtypes, denoted as H1 to H16, and 9 NA subtypes, denoted as N1 to N9 [4]. Based on the virus pathogenicity in chicken, IAVs are classified into highly and low-pathogenic IAVs (HPIAVs and LPIAVs), respectively. LPIAVs include all subtypes, whereas HPIAVs include some of the H5 and the H7 subtypes [5].

Wild birds, particularly of the Anseriformes and Charadriiformes orders, serve as the natural reservoirs of IAVs, where these viruses typically circulate asymptomatically [6,7]. HPAI viruses, specifically of the H5 subtype, pose a significant threat to both poultry and human health. In 1996, in a domestic goose in Guangdong China (Gs/GD), the lineage of (HPAI) A(H5N1) viruses was initially detected. Since then, these viruses have continued to circulate and spread, with their hemagglutinin (HA) genes diversifying into multiple genetic clades. The H5 Gs/GD lineage clade 2.3.4.4, particularly the H5N8 subtype, was first identified in domestic poultry in China in 2010. By 2014, it had caused numerous outbreaks in domestic and wild birds in South Korea, Japan, China, Europe, and North America [8]. Two distinct clusters of HPAI A(H5N8) viruses emerged during these outbreaks: group A viruses were detected in China, South Korea, Japan, Taiwan, Canada, the United States, and Europe (Buan-like), while group B viruses were identified only in China and South Korea in 2013–2014 and have since spread globally (Gochang-like virus) [9]. In 2016/2017, a novel reassortant virus of subtype H5N8 within clade 2.3.4.4 was discovered in wild birds at the Russian–Mongolian border during the summer of 2016. This virus subsequently spread to numerous countries across Europe, Asia, and Africa, including Egypt in the autumn of 2016 [10,11]. Some strains of H5N8 displayed decreased virulence, lower spreading ability, and a longer mean death time compared to the original HPAI strain of H5N1. The virus caused high death rates in both wild and domestic birds and crossed species boundaries, infecting mammals like humans, foxes, and seals in multiple countries [12,13,14]. In recent times in Europe, the EuroII-2020 subclade, belonging to clade 2.3.4.4b, has become the most prevalent H5N8 variant. This subclade is has been closely connected to counterparts identified in Iraq, Russia, and Kazakhstan since May 2020. Additionally, diverse genotypes have emerged through multiple reassortment events with LPAI viruses found in wild birds across Eurasia [15].

Egypt is considered a hotspot for IAVs owing to its geographical positioning in the convergence of two migratory bird flyways via the Mediterranean-Black Sea and East Africa–West Asia flyways which overlap with the more regional Rift Valley–Red Sea flyway and the endemicity of different IAV subtypes in bird populations [16]. In late 2016, HPAI H5N8 clade 2.3.4.4b virus was initially detected in migratory avian species, particularly common coot and green-winged teal, in Egypt [11]. A comprehensive surveillance initiative launched from late 2016 to 2021 revealed six distinct genotypes of the HPAI H5N8 virus in both migratory and domestic avian populations. The rapid transmission of the virus within domestic poultry across different regions in Egypt posed a significant threat to the poultry industry [17]. By 2021, a subclade called EuroII-2020 H5N8 was discovered in ostriches in Egypt [18]. Various H5 subtypes, including H5N1 and H5N5, all belonging to clade 2.3.4.4b, were identified in wild birds in Egypt [19,20]. A recent study indicated that H5N8 viruses from Egypt also belong to clade 2.3.4.4b and exhibit close genetic relationships with strains from China, Iran, and Iraq, sharing over 98% nucleotide-sequence similarity. The pathogenicity of these H5N8 strains has been confirmed through multiple assays, demonstrating a high risk to domestic birds, even those that have been vaccinated [21].

Another recent finding suggested that H5N8 can adapt to mammalian hosts through specific genetic markers, which enhance its growth and virulence in these species. A comprehensive model demonstrated that as mutations accumulate, the risk score for mammalian adaptation increases significantly. Notably, a strain isolated from migratory birds exhibited high pathogenicity in mice, with a lethal dose indicating severe effects on multiple organs. Although H5N8 primarily circulates among birds, its adaptability and emerging evidence of mammalian infections underscore the need for heightened monitoring and research to address potential zoonotic threats [22].

The current study focused on the genome sequence analysis of 17 H5N8 viruses isolated from duck farms and backyards. The primary objective was to identify key genetic signatures (KSs) associated with an increased risk of a human pandemic. These potential signatures include modifications affecting host specificity, virulence, replication capability, transmissibility, or drug susceptibility. The present study also aimed to enhance our understanding regarding the genetic attributes and origins of the recently emerged highly pathogenic avian influenza (HPAI) H5N8 viruses in Egypt, along with exploring their genetic and phylogenetic relationships.

## 2. Materials and Methods

### 2.1. Samples and Study Area

The present study was performed during the winter season 2021/2022 on 100 duck farms located in four governorates within the delta region of Egypt. These farms consisted of 60 breeder farms and 40 small and backyard farms. To determine the presence of avian influenza, samples were collected under aseptic conditions from ducks suspected of being infected. These samples included oropharyngeal and cloacal swabs, as well as tissue specimens from the liver, pancreas, spleen, brain, tracheas, and lungs. The collection of these samples was performed carefully as per the guidelines established by the World Health Organization. To form a single working sample per farm, individual bird samples from 10 ducks were combined from each farm (10 ducks/flock/1 pool/1 sample). The birds, ranging in age from two weeks to one year, were promptly transported to the laboratory following their demise. Virus isolation, real-time RT-qPCR, and biopsy were conducted at the Animal Health Research Institute’s Reference Laboratory for Veterinary Quality Control on Poultry Production. In Table S1, epidemiological information regarding samples that were taken and the samples positive for H5N8 avian influenza is detailed. Table S2 summarizes the genotyping of Egyptian IAV H5N8 genes analyzed in the current study with their accession numbers in GenBank.

### 2.2. Virus Detection and Isolation

The process of detecting and identifying viruses was performed using real-time RT-qPCR. The sample was prepared for analysis by extracting the viral RNA in accordance with the QIAamp viral RNA mini kit’s (Qiagen, GmbH, Hilden, Germany) instructions. A 25 µL final volume of the QuantiTect RT Mix was employed to perform real-time RT-PCR amplification in accordance with the instructions provided with the reagent. Metabion (Germany) supplied the primers and probes utilized in the subtyping of H5N8 by means of real-time RT-qPCR. These components were derived as previously described by Domingo, E. [23]. Samples were suspended in phosphate-buffered saline (PBS; pH 7.2) treated with antibiotics. These suspensions were then inoculated into 9–11-day-old specific-pathogen-free (SPF) embryonated chicken eggs, which were incubated at 37 °C for 4–5 days. The presence of avian influenza virus (IAV) in the allantoic fluid was detected using a hemagglutination (HA) assay with chicken erythrocytes [24].

### 2.3. cDNA Synthesis

In this analysis, viral RNA was extracted from allantoic fluid using the QIAamp Viral RNA Mini kit’s (Qiagen, GmbH, Hilden, Germany), following the manufacturer’s protocol. Once extracted, the viral gene-positive RNA was reverse-transcribed to generate complementary DNA (cDNA). For this step, the RevertAid First Strand cDNA Synthesis Kit (Thermo Fisher Scientific, Waltham, MA, USA) was used, ensuring efficient and accurate cDNA synthesis from the RNA template.

### 2.4. Sequence Analysis of IAV Genes

The nucleotide sequences of all IAV gene segments were determined by nanopore sequencing using a MinION Mk1B (Oxford Nanopore Technologies, Oxford, UK), as previously described [25,26]. Briefly, viral genes were amplified from synthesized cDNA by KOD One PCR Master Mix Blue (TaKaRa Bio Inc., Otsu, Japan) with gene segment-specific and subtype-specific primer sets [27]. PCR amplicons were purified using a Wizard SV gel and PCR clean-up system kit (Promega, Madison, WI, USA). Adapter ligation was performed using a direct cDNA Sequencing Kit (Oxford Nanopore Technologies) with a Native Barcoding Expansion Kit (Oxford Nanopore Technologies). Sequencing was processed using a Flongle flow cell (Oxford Nanopore Technologies). The consensus sequences for each gene segment were generated using Geneious Prime v.2021.1.1 (Biomatters Ltd., Auckland, New Zealand).

### 2.5. Mutational Analysis of Genes

The gene mutations in Egyptian H5N8 viruses were analyzed utilizing computational tools and approaches. Nucleotide homology and protein sequences were analyzed using the program MegAlign (DNAStar, Madison, WI, USA). Glycosylation sites of NA and HA were determined using the NetNGlyc 1.0 Server (http://www.cbs.dtu.dk/services/NetNGlyc/) (accessed on 30 April 2023), and mutations at antigenic and receptor-binding sites were identified through alignment of genes from Egyptian isolates. FluSurver (https://flusurver.bii.a-star.edu.sg/) (accessed on 30 April 2023). was employed to detect mutations in influenza virus proteins that have the potential to impact a multitude of biological attributes, such as drug resistance, virulence, host specificity, and others. Additionally, we used the CDC inventory to annotate amino acid positions by comparing them with the 390 A/Vietnam/1203/2004 reference and the original A/H5N1 goose/Guangdong reference from 1996 [28].

### 2.6. Phylogenetic Analysis

The consensus sequences were aligned using the MAFFT v7 online server (https://mafft.cbrc.jp/alignment/server/) (accessed on 30 April 2023).manually assembled using the BioEdit software package version 5.0.9 [29]. The open-source BLAST program (National Center for Biotechnology Information, Bethesda, MD, USA, http://blast.ncbi.nlm.nih.gov/Blast.cgi) (accessed on 30 April 2023), was used to identify the closest related sequences and analyze genetic similarities. The consensus sequences were then compared with sequences from the most closely related virus strains available in the GISAID and NCBI databases. Maximum-likelihood phylogenetic trees for each gene segment were generated using IQTREE v1.6.6 (https://github.com/iqtree/iqtree1) (accessed on 30 April 2023), with the best-fitted model selected dependent on the Akaike criterion: (TIM + F + G4) for HA, (K3Pu + F + R2) for NA, (K3P + G4) for M, (K3Pu + F + G4) PA, (TIM + F + G4) NP, (TN + F + G4) for PB1, (GTR + F + G4) for PB2, and (TPM2u + F + G4) for NS. Their robustness was assessed through ultrafast bootstrap resampling analysis with 1000 replications [30,31]. The resulting phylogenetic trees were visualized using FigTreev1.4.4 software (http://tree.bio.ed.ac.uk/software/figtree/) (accessed on 30 April 2023).

### 2.7. Genotype Analyses

To understand the genetic diversity of influenza A viruses (IAVs) in Egypt, the analysis involved defining clusters for each gene segment based on specific criteria. These criteria included sharing a phylogenetic cluster with a minimum bootstrap value of 70 and sequences having more than 97% nucleotide-sequence identities. Each genotype was determined by the combination of the cluster assignment of eight gene segments.

## 3. Results

### 3.1. Clinical Signs and Pathological Examination

**Figure 1:**
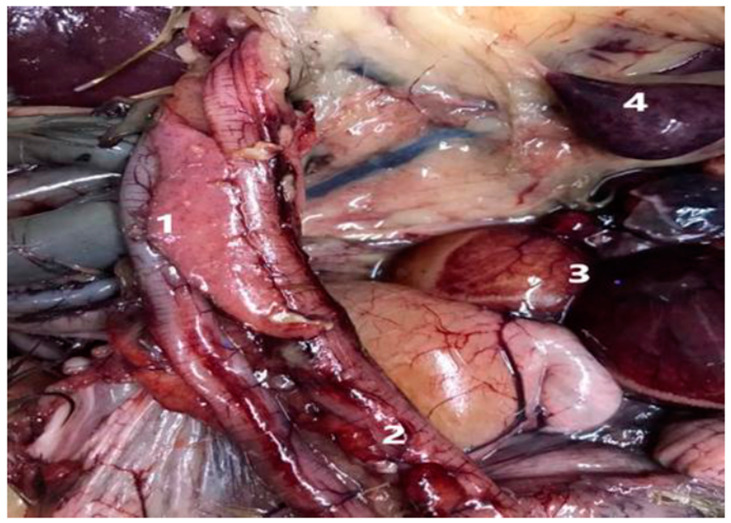
Pathological outcomes in the suspected case 1. Pancreatitis with petechial hemorrhage in pancreas. 2. Duodenitis. 3. Congested ovary and oviduct 4. Necrotic foci in spleen with congestion.

The bird populations exhibited symptoms and physical characteristics that suggested the presence of avian influenza, including a lack of appetite, reduced egg production in vaccinated farms, discharge from the eyes and nose, depression, greenish-white diarrhea, and varying degrees of high death rates. Some birds that were not vaccinated displayed nervous signs such as full reluctance to move, prostration, wing paralysis, opisthotonus, torticollis, and abnormal gait. The main postmortem lesions were generalized congestion, encephalitis, tracheitis, pneumonia, degeneration and multifocal necrosis in the liver, multifocal necrotic pancreatitis, and petechial hemorrhage in the pancreas, duodenitis, congested ovaries, and oviduct and necrotic foci in spleen with congestion (Figure 1).

### 3.2. Virus Detection and Isolation

From a total of 100 samples collected from duck farms and backyards, only 26 tested positive for HA activity and M gene real-time PCR (RT-PCR). Of these 26 H5N8-positive samples, 17 were successfully isolated on embryonated chicken eggs (ECEs). Whole-genome sequencing was then performed on these 17 H5N8-positive specimens (Tables S1 and S2).

### 3.3. Genetic Analysis of the Viral Genome of Egyptian H5N8 Viruses

In the HA protein, twenty-two viruses in this study share a common and characteristic polybasic cleavage site motif “PLREKRRKR/GLF”, which is typical of Egyptian H5N8 viruses isolated from 2016 to 2022. The presence of a polybasic cleavage site is a significant virulence determinant and is associated with the highly pathogenic avian influenza (HPAI) phenotype. This motif is crucial in the virus’s ability to cause severe disease and is a key factor in its pathogenicity [32].

Comparing the HA protein of our Egyptian H5N8 viruses with Flusurver reference strain A/Sichuan/26221/2014 (H5N6) revealed significant evolutionary mutations. These mutations have altered the receptor-binding and antigenic binding sites of the HA protein. Mutations such as T110S, T139H, N205T, T139P, A172V, N199T, and R512K are found in critical regions that interact with terminal sialic acids on the surface glycans of host cells. These regions are essential for receptor binding, and alterations in these sites can significantly impact how the virus recognizes and binds to host cell receptors. Understanding these changes is important for predicting shifts in host range and could aid in assessing the risk of cross-species transmission (Table 1 and Table 2) [33].

Although analysis of Egyptian H5N8 viruses showed no deletions in the neuraminidase (NA) genes, there are several mutations that affect NA drug sensitivity and evade antiviral treatments (Table 1 and Table 2). Additionally, several amino acid changes were observed in two regions that are considered crucial for NA hemadsorption activity. In particular, two isolates, namely, F653/18 and F653/32, showed ^361^RTISRNSRSGFE^372^, and five other isolates, namely, F653/10, F653/12/, F653/13, F653/23, and F653/24 showed ^390^RQVVVDDLNWSGS^404^ (Table S3).

The NA protein of the analyzed viruses showed conserved N-linked glycosylation sites at positions 54 (NETV), 67 (NTSV), 84 (NNTE), 144 (NGTV), and 398 (NWSG) (Table S4). Notably, all analyzed viruses lacked a glycosylation site at position 293 (NWTG), which is also in line with observations in the EuroII-2020 subclade and Egy/ostrich/2021 viruses [18]. Two new possible glycosylation sites were recognized in isolates of the current study; a glycosylation site at position 2 (NPSQ), which was found in nine isolates, and a glycosylation site at position 54 (NETA) that was found in one isolate (Table S4). 

A series of notable mutations were identified in both the HA gene (54D, 236N, 269V, and 306V) and the NA gene (191V, 213V, and 342H). Furthermore, all isolates demonstrated mutations in PB2 (292I, 615I, and 714S), PB1 (211R, 431H, and 691K), and PB1-F2 (42W and 46S). Other proteins exhibited mutations as well, including PA (96N, 120I, 214L, 348I, and 445Y), NP (318S), M1 (42L, 134R, 209A, and 248I), and M2 (18K). In addition, distinct variations were detected in NS1 (100R, 210G, and 215S) and NS2 (28V). These mutations were newly identified and had not been previously reported in Egyptian strains from 2020 to 2021 (Table S5).

The comparison of protein in Egyptian viruses to the reference strain as suggested by FluSurver revealed a series of mutations that have emerged over time. These mutations impact virulence, shifts in host specificity, and interactions with host proteins (Table 1 and Table 2).

Further analysis was performed to investigate other virulence and mammalian adaptation factors in the viral PB2, PB1, PA, NP, M2, NS1, and NS2 proteins (Tables S6 and S7).

### 3.4. Phylogenetic Analysis and Sequence Similarity

HA gene analysis revealed two distinct genetic groups (I and II), with group II further subdivided into G1-II (evolved from EuroII-2020) and G2-II (from Egypt Genotype 6), suggesting a mix of local and imported viral strains. 

**Figure 2:**
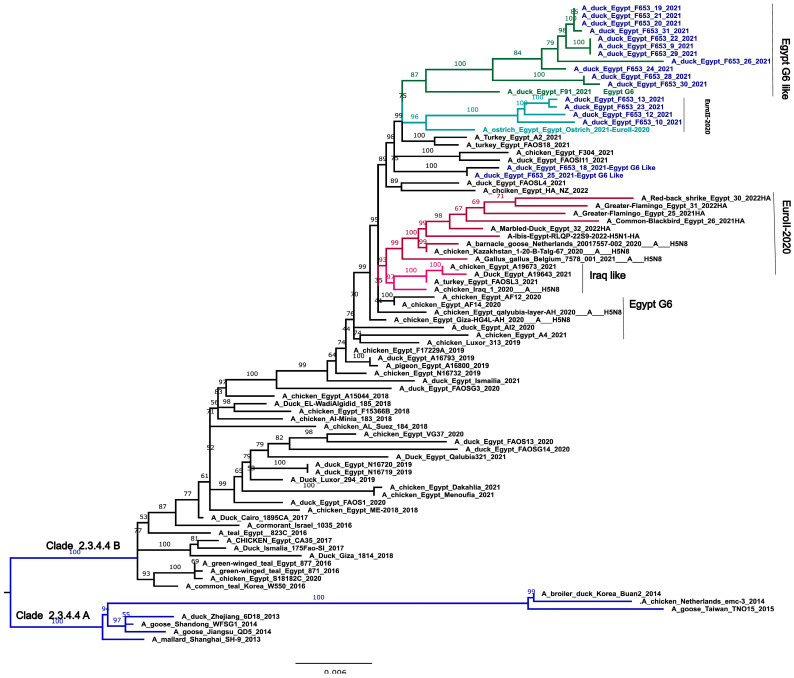
Phylogenetic trees of the nucleotide sequences of the HA gene segments of the avian influenza H5 subtype. Maximum-likelihood calculations were performed using the IQ-TREE v 2.1.3 software with the best fit model according to the Bayesian criterion (TIM + F + G4). Egyptian HPAI H5N8 viruses used in this study are colored in blue. Blue branches may indicate clade 2.3.4.4A, Red branches may indicate EuroII-2020 subclade, light green indicate viruses originated from EuroII-2020 subclade and deep green indicate viruses originated from Egypt G6.

**Figure 3:**
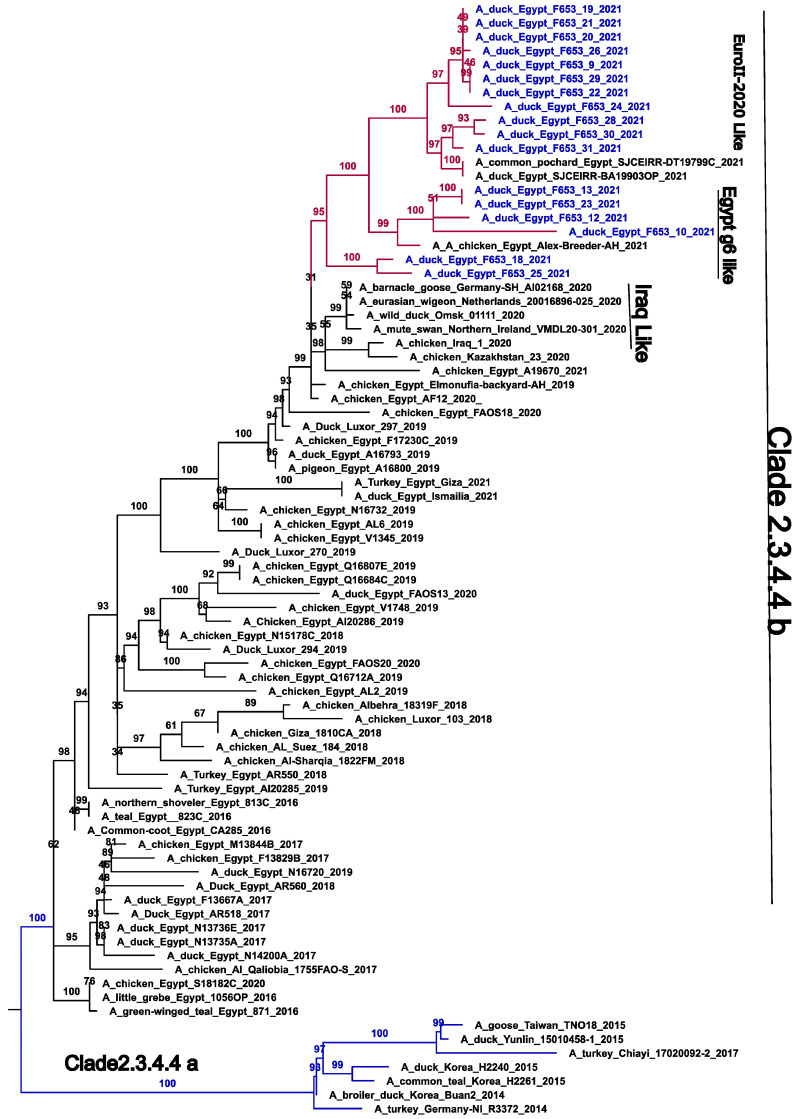
Phylogenetic trees of the nucleotide sequences of the NA gene segments of the avian influenza H5 subtype. Maximum-likelihood calculations were performed using the IQ-TREE v 2.1.3 software with the best fit model according to the Bayesian criterion (TIM+F+G4). Egyptian HPAI H5N8 viruses used in this study are colored in blue. Blue branches may indicate clade 2.3.4.4A.

However, in the NA gene, genetic group I is subdivided into three subgroups with a different evolutionary origin: G1 (I and II), which evolved from Egypt Genotype 6, and G1-III, which evolved from the EuroII-2020 subclade. The long branch length between the genetic group and its ancestors (EuroII-2020 subclade) suggests that these genetic groups may have been circulating undetected in wild or domestic birds, thus indicating potential local transmission prior to detection. These contrasting genetic patterns highlight the complexity and potential reassortment events between the EuroII-2020 subclade and Genotype 6 in Egypt during 2019–2021 (Figure 2 and Figure 3).

**Figure 4:**
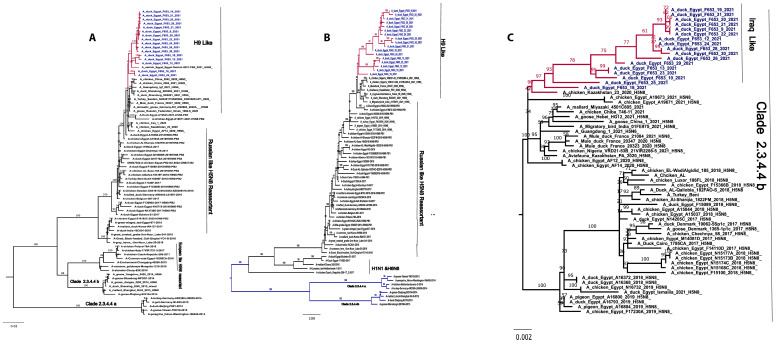

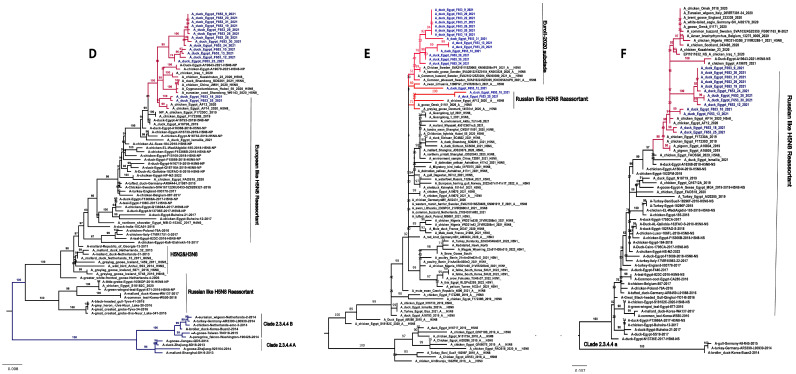
Phylogenetic tree of internal genes. The PB1 and PB2 genes of all Egyptian strains cluster in H9-like viruses and PA genes are related to A/chicken/Kazakhstan/23/20209 Iraqi-like viruses (**A**) Phylogenetic tree of the pb2 gene. (**B**) Phylogenetic tree of the PB1 gene. (**C**) Phylogenetic tree of the PA gene. Egyptian HPAI H5N8 viruses used in this study are colored in blue. The NP related to European-like H5N8 reassortant 2016 and European-like H5N8 reassortant 2020, M related to Russian-like H5N8 reassortant 2016 and Russian reassortant H5N5 2021, and NS closely related to Russian reassortant-like H5N8 2016. (**D**) Phylogenetic tree of the NP gene. (**E**) Phylogenetic tree of the M gene. (**F**) Phylogenetic tree of the NS gene. Egyptian HPAI H5N8 viruses used in this study are colored in blue.

The PB2 and PB1 gene sequences of the isolates showed a nucleotide-sequence homology ranging from 98% to 100% and 97% to 99%, respectively. In the case of PB2 and PB1, they are related to H9-like viruses (Figure 4). 

The PA gene sequences of the isolates showed 98-100% nucleotide homology and formed distinct genetic groups originating from chicken/Kazakhstan/23/2020 H5N8 (Iraqi-like viruses). The long horizontal branch lengths observed between these PA gene groups and their closest relatives suggest that the virus may have been circulating undetected in wild or domestic birds before it was introduced into Egypt (Figure 4).

The M gene sequences of the isolates showed 98-100% nucleotide homology, and phylogenetic analysis revealed three distinct genetic groups (I, II and III) within clade 2.3.4.4b. Each group evolved independently from different strains, including EuroII-2020 strains, Russian reassortant H5N5 2021-like, shelduck/Kalmykia/1814-1/2021 (H5N5), and A/Dalmatian pelican/Astrakhan/417-1/2021 (H5N5). Group I, considered an outgroup, mainly originated from Egyptian endemic Russian-like H5N8 reassortant strains. The NS gene sequences of the isolates showed 97-100% nucleotide homology and formed distinct genetic groups originating from Russian reassortant-like H5N8 2016 (Figure 4).

The NP gene sequences of the isolates showed 98-100% nucleotide homology, forming three distinct genetic groups (I, II and III). Group I is associated with European H5N8 reassortant strains from 2016, while groups II and III are linked to European H5N8 reassortant strains 2021 (Figure 4).

BLAST searches on the NCBI platform for all eight genes revealed that our H5N8 viruses exhibit close genetic relationships with strains from China, India, Kazakhstan, and Iraq, sharing over 98% nucleotide-sequence similarity (Table 3 and Table 4).

## 4. Discussion

Since 2016, H5N8 viruses in clade 2.3.4.4.b have been spreading across Egypt [17,18,19,20,21,22,23,24,25,26,27,28,29,30,31,32,33,34,35,36,37,38,39,40,41,42,43,44,45,46,47,48,49,50,51,52,53,54,55,56,57,58,59,60,61,62,63,64,65,66,67,68,69,70,71,72,73,74,75,76,77,78]. Previous reports indicated that the major genotypes of H5N8 avian influenza viruses (IAVs) in Egypt can be divided into six distinct genotypes [17,51]. Recent studies have also reported the introduction of the EuroII-2020 subclade, particularly found in ostriches [18]. 

**Figure 5:**
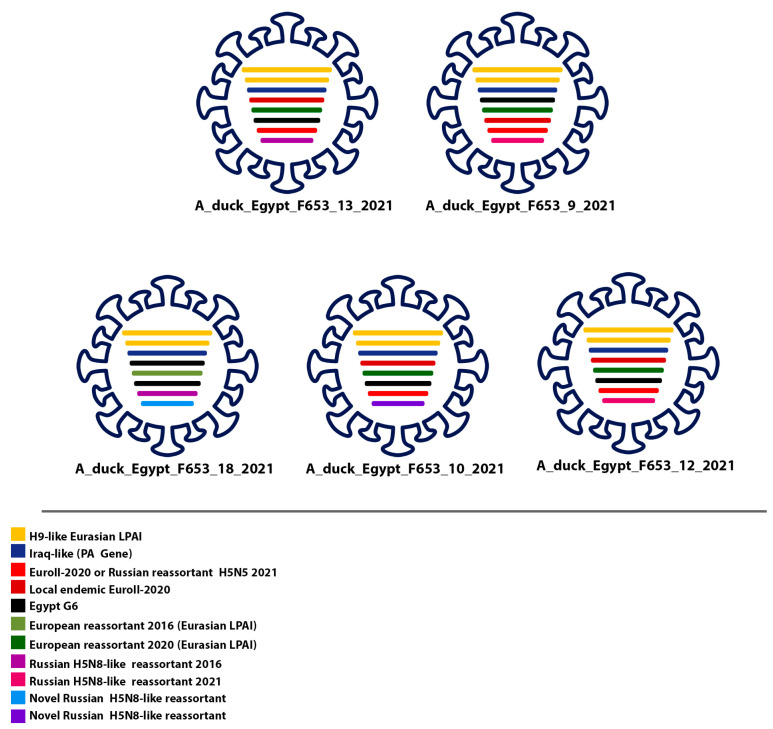
Representation of the newly identified genotypes of H5N8 viruses in poultry in Egypt. The newly identified genotypes of H5N8 viruses in poultry in Egypt are thought to be a result of genetic contributions from various sources, including Iraqi-like viruses (Kazakhstan/PA/2020 (H5N8), EuroII-2020 subclade, Eurasian LPAI, and Egypt G6. Notably, the NS gene differs in A/duck/Egypt/F653/10/2021, A/duck/Egypt/F653/12/2021, and A/duck/Egypt/F653/13/2021, while the other segments exhibit a high degree of similarity. See also Table 4.

In this study, molecular and phylogenetic analyses of H5N8 strains found in Egypt revealed a significant link to waterfowl-associated strains from Europe. Our findings identified two distinct types of low-pathogenic avian influenza (LPAI) viruses in the region. The first type includes locally endemic reassortant viruses, such as the H5N8 reassortant 2016 and Russian-like H5N8 reassortant 2016, derived from European sources. These viruses have established themselves in the local population since 2016, demonstrating persistence and integration with existing endemic viruses, highlighting their adaptation and stability within the local environment. The second type comprises recently introduced viruses from Eurasia, such as H9-like strains, representing new genetic variants that were not previously detected locally, indicating ongoing viral introductions and growing genetic diversity (Figure 5).

The evolution of the EuroII-2020 and endemic Genotype 6 strains has led to the emergence of new genotypes resembling these original strains. Furthermore, reassortment events have occurred between these newly evolved genotypes, the recently introduced Eurasian viruses—including H9-like strains—and the endemic Russian-like and European-like reassortants from 2016. Afterward, Egypt witnessed the emergence of five distinct genotypes of the H5N8 avian influenza virus, displaying a similar genome constellation where the internal genes (NP, PB1, PB2) were derived from Eurasian low-pathogenic avian influenza viruses (LPIAVs). In contrast, the M, NS, and PA genes were sourced from highly pathogenic avian influenza (HPAI) viruses. However, a recent study has identified a new genome constellation where the majority of genes are from LPIAVs, with exceptions for the HA, NA, and NS genes. This shift reflects an evolving genetic landscape in the H5N8 virus [77]. 

The whole-genome analyses of HPAI H5N8 viruses indeed revealed several key mutations (T156A, S123P, D183N, and S223R) that could influence the virus’s adaptation, persistence, and virulence. These mutations were found to be associated with viruses from the 2.3.4 clade, which contained particular residues (T156A, S123P, D183N, and S223R) and exhibited transient persistence before implementing a compensatory mechanism that entailed a modification in the NA protein. This suggests that while these HA mutations initially provided some advantage, the viruses required additional changes in the NA protein to achieve stable adaptation and persistence [78]. In our study, we identified novel alterations in the amino acid sequences of the neuraminidase hemadsorption site which could impact the enzyme’s catalytic efficiency. The hemadsorption site is essential for NA function and plays a key role in interacting with HA receptor-binding specificity. Novel mutations in NA, combined with changes in HA, may be crucial for the emergence of pandemic influenza viruses. This underscores the importance of NA in viral evolution and adaptation [79].

Recent whole-genome analyses of the HPAI H5N8 viruses have revealed molecular mutations that could potentially increase their zoonotic potential. The H5 protein of all H5N8 viruses described here carries the mutations S133A, D94N, S154N, T156A, and V182N (H5 numbering). In addition, the H5 protein of the H5N8 virus A/duck/Egypt/F653/12/2021 features K189R/T mutations. These mutations in the H5 protein have been shown to increase binding to alpha2,6-SA. The substitution K189T in the HA protein, which is transported by Egyptian strains, can be utilized to evaluate the potential pandemic risk throughout surveillance of emerging viruses [38]. Furthermore, it is important to note that the S133A mutation characterizes the HA gene of all H5 viruses identified in Europe since early 2021 [34,80]. Moreover, the substitution T156A significantly modifies the antigenic characteristics of the 2.3.4 H5 viruses. Notably, T156A is consistently detected in airborne transmission-capable mutants adapted to ferrets. While most H5N1 viruses typically have a glycosylation site at positions 154–156, the T156A mutation leads to the loss of this glycosylation site. The loss of this glycosylation site is associated with an increased ability of the virus to infect mammals, suggesting that the loss of this glycosylation site enhances the virus’s adaptability and virulence in mammalian hosts [81]. In this investigation, it was determined that the HA protein of all our H5N8 Egyptian viruses contains Q222 and G224 residues (H5 numbering). These residues signal a preference for avian-like receptors, which reduces the likelihood of human-to-human transmission [82]. Additionally, the presence of these residues contributes to an increased proinflammatory response [34]. 

The N30D, I43M, and T215A substitutions in the M gene, which are noted in all isolates, have been associated with increased virulence in animal models [42]. The CCHH zinc-finger motif within helix 9 of the M1 protein is believed to play a crucial role in regulating virulence, particularly in mice [83]. 

Notably, there are mammalian adaptive markers present in the PB2 protein of thirteen H5N8 viruses, including L89V, G309D, T339K, R477G, I495V, K627E, and A676T. The L89V substitution in the PB2 protein is located in the vicinity of residues 51–259, which is implicated in interactions with PB1, and residues 1–515, which is the region accountable for binding to heat shock protein 90 (HSP90) [84]. Furthermore, substitutions G309D, T339K, R477G, and I495V have been identified in the region where HSP90 binds. By facilitating interactions between polymerase subunits or between the polymerase and host factors, these substitutions increase polymerase activity and, consequently, mammalian virulence [85].

It is noteworthy that in publicly available sequences, a combination of these six mutations along with 627E in PB2 was detected in 58 out of 340 avian influenza viruses (IAVs) responsible for human infections, including H5N1, H7N7, and H9N2. Moreover, a substantial proportion (67.5%, 13,074 out of 19,338) of influenza A viruses (IAVs) isolated from avian hosts possess this combination of mutations in PB2. Indeed, prior reports have highlighted that numerous low-pathogenic avian influenza viruses (LPIAVs) isolated from wild birds, which exhibit a combination of six specific mutations, can replicate in the BALB/c mouse model without the need for prior adaptation [86]. In accordance with our study, the substitutions L89V, G309D, and T339K were noted in Egypt H5N1 viruses recently isolated [87]. Moreover, the K497R substitution in the PA protein of these seventeen H5N8 viruses is particularly significant due to its pivotal role in facilitating the adaptation of avian viruses to mammalian hosts. Furthermore, it is worth noting that the pathogenicity and host specificity of influenza viruses can be directly influenced by the K615R substitution in the PA gene [85]. Also, in the PB1 protein, the D3V and D622G substitutions are associated with increased viral replication and enhanced polymerase activity, both in mammalian and avian cell lines. These mutations also correlate with elevated polymerase activity and heightened virulence in rodents. Additionally, the P598L mutation in PB1 is known to further augment polymerase activity in mammalian cells and rodents [46]. This modification has been documented to enhance the polymerase activity of a weakened human influenza virus containing the PB2 K627E mutation, which is typically involved in host adaptation and virulence enhancement in mammals. These mutations contribute to the virus’s ability to replicate more efficiently and potentially increase its pathogenicity across species [46]. 

Surprisingly, the NS1 protein in all Egyptian strains of these study lacks a PDZ motif and contains residues F103 and M106 within the cleavage and polyadenylation specificity factor (CPSF30) binding site [88]. These residues, which are frequently found in human isolates, inhibit the production of beta interferon (IFN-β) mRNA and stabilize CPSF30 binding [89]. 

Similar to a previous study in Egypt, numerous mutations were found in different viral proteins, and mammalian adaptations and virulence-related characteristics were observed in current Egyptian H5N8. These mutations were detected in PB2 (588V, 598T, and 504V), PA (127V, 550L, and 672L), PB1 (13P), NP (398Q), NS2 (31I), and NS1 (42S and 189N). The mutations identified in key viral proteins, especially in PB2 and PA, suggest an increased ability of the virus to adapt to mammalian hosts, potentially posing a higher risk for human infections. Despite these genetic characteristics, no proof of human infection with these viruses was found [17]. 

In conclusion, domestic ducks play a significant role as intermediaries in the transmission of avian influenza, bridging migratory waterfowl and terrestrial poultry. As asymptomatic carriers, they can spread the influenza A virus (IAV) to other species, underscoring the need for improved surveillance and management strategies.

To effectively control avian influenza in Egypt, it is crucial to enhance monitoring of (LPIAVs) in domestic ducks and poultry. Targeting specific strains that drive virus evolution and reinforcing biosecurity measures on farms and in live-poultry markets are essential steps.

Education of poultry farmers and workers on best practices, alongside collaboration with international organizations, will facilitate the exchange of information and strategies. Investment in research to understand virus reassortment and the development of new vaccines and preventive measures will be vital for managing outbreaks and mitigating risks.

A comprehensive strategy must include ongoing monitoring of genetic changes, evaluation of virological attributes, and strengthened surveillance initiatives. Updating vaccine seeds to address antigenic mismatches with circulating strains is recommended. Consistent vaccination efforts, particularly in backyard ducks, are necessary to overcome challenges related to vaccination failures. Adherence to rigorous biosafety and biosecurity measures is critical to safeguard poultry health and prevent future outbreaks of HPAI.

## Figures and Tables

**Table 1:** Amino acid markers associated with specific phenotypic effects found in the genome of HPAI H5N8 viruses from Egypt sequenced in the current study by comparing them with the 390 A/Vietnam/1203/2004 reference and the original A/H5N1 goose/Guangdong reference from 1996.

Protein	Amino Acid Change(s) H5 Numbering	Detections in Egypt	Phenotypic Consequences	Ref
HA	D94N	Yes	Increased virus binding to α2–6	[33]
	S133A	Yes (17/17) 100%		[34]
	S154N	Yes (17/17) 100%		[35]
	T156A	Yes (17/17)		[36]
	V182N	Yes (17/17) 100%		[37]
	K189R/T	Yes (1/17) K189T		[38]
	S107R, T108I	Yes (17/17)		[39]
	323 to 330 (R-X-R, K-R)	Yes PLREKRRKR/GLF	HPAI cleavage site	[40]
NA	I314V	Yes (17/17)	Reduced susceptibility to oseltamivir	[41]
M	N30D	Yes (17/17)	Increased virulence in mice, chickens, and ducks	[42]
	I43M	Yes (17/17)		
	T215A	Yes (17/17)		
PB2	I292V	Yes (17/17)	Increased polymerase activity	[43]
	K389R	Yes (17/17)		[44]
	A588V	Yes (2/17)		[45]
	V598T/I	Yes (17/17)		[44]
	K627E	Yes (17/17)	Increased virulence in chickens	[46]
	S715N	Yes (17/17)	Decreased virulence in mice	[47]
	L89V, G309D	Yes (17/17)	Increased polymerase activity	[48]
	L89V, G309D, T339K, R477G, I495V, K627E, A676T	Yes (13/17)		[48]
PB1	D3V	Yes (17/17)		[49]
	D622G	Yes (17/17)		[50,51]
	P598L	Yes (17/17)		
PB1-F2 length	Truncation	Truncation 52 lengths	Affects viral dissemination, pathogenesis, and transmission	[52,53,54,55,56,57]
PA	D55N	No (0/17)	Host specificity marker through statistical methods (D in avian, N in human)	[58]
	S37A	Yes (17/17)	Increased polymerase activity	[59]
	P190S	Yes (17/17)	Decreased virulence in mice	[60]
	N383D	Yes (17/17)	Increased polymerase activity	[61]
	N409S	Yes (17/17)		[59]
	K497R	Yes (17/17)		[62]
NP	M105V	Yes (17/17)	Increased virulence in chickens	[63]
	A184K	Yes (17/17)	Increased replication in avian cells and virulence in chickens, enhanced IFN response	[64]
NS	P42S	Yes (17/17)	Increased replication in mammalian cells, decreased interferon response	[65]
	I106M [I101M]	Yes (17/17)		[66]
	C138F	Yes (17/17)		[67]
	V149A	Yes (17/17)	Increased virulence and decreased interferon response in chickens	[68]
	L103F, I106M [L98F, 101M]	Yes (17/17)	Increased virulence in mice	[69]
	222–230 deletion	Yes (17/17)	Increased replication in mammalian and avian cell lines	
	P87S	Yes (17/17)	Host specificity marker through statistical methods (S in human, P in avian)	[70]

**Table 2:** Comparative analysis of amino acid markers linked to phenotypic traits in HPAI H5N8 viruses from Egypt: insights from current genome sequencing and Flusurver reference.

**Flusurver Reference**	**Mutation**	**Phenotypic Effect**	
HA	A/Sichuan/26221/2014 (H5N6)	T110S, T139H, N205T, T139P, A172V, N199T, and R512K	Influence receptor recognition and potentially shift host specificity
		N110S	Host specificity shift Modifies T-cell epitope presented by MHC molecules Antibody recognition sites
		N205T, G16S, and I214V	Virulence Host cell receptor binding Antibody recognition sites Modify T-cell epitope presented by MHC molecules binding host protein(s)
		A99V, A102V, T156A, I178M, and A201E, E284G, M285V, K492E, E495A, and P505T	Antibody recognition sites
		A99N	Mild drug resistance and antibody recognition sites
NA	A/Baikalteal/KoreaDonglim/3/2014 (H5N8)	D220H, A245S, T295M, N396D, S397L, and N396D	Antibody recognition sites Strong and mild drug resistance
		T265A	Strong drug resistance
		Y450H	Binding host protein(s)
		N46K, T295M, and T48A	The removal of N-acetylneuraminic acid (NA) glycosylation can increase the virulence and pathology of influenza A viruses in birds and mice [71,72]
		V106I and T329A	Mild drug resistance
NP	A/duck/HongKong/24/1976 (H4N2)	T396N, A423V, and A423T	Modify T-cell epitope presented by MHC molecules
PA	P A/Netherlands/219/2003 (H7N7)	K113R, S184N, S224A, and P653S	Modify T-cell epitope presented by MHC molecules
		K615R	Host specificity shift, virulence
PB2	A/mallard/Astrakhan/263/1982 (H14N5)	V495A, A588V, T676A, I676A, and V553I	Host specificity shift, virulence
		E558D	Modify T-cell epitope presented by MHC molecules
NS1	A/chicken/BCFAV8//2014 (H5N2)	G70K and I81V	Host specificity shift
		E227G, G232R, S205N, and R231stop	Virulence
		S7L, H17Y, S48T S114P, T143A, L166F, D189N, and Q218stop	Binding host protein
NS2	A/WSN//1933 (H1N1) and A/quail/Italy/1117/1965 (H10N8)	M19L and A48T	Virulence
NS1	A/Canada/720/2005 (H2N2)	M81V, S205N, Q218stop, R227G, K229E, and V230I	Virulence
		S7L, H17Y, Q21R V22F, R118K, T143A, L166F, N171D, and D189N	Binding host protein
		T215S and T215P	Host specificity shift, virulence

**Table 3:** Nucleotide-sequence homology comparison of the gene segments of HPAI H5N8 viruses from Egypt with available sequences in NCBI platform.

	Accession No.	Closest Relative	Identity (%)
PB2	EPI1902241	A/chicken/Egypt/F17230B/2019 (H5N8)	99%
	ON862701.1	A/duck/Shandong/SD0263/2021 (H5N6)	98.6%
	EPI1814302	A/goose/Omsk/30006/2020 (A/H5N8) segment 1 (PB2)	98%
	EPI1811685	A/goose/Russian Federation/Kurgan/1345-25/2020 (A/H5N8) segment 1 (PB2)	98%
	ON329039.1	A/chicken/China/2042/2020 (H9N2)	98.6%
PB1	MT261707.1	A/duck/Egypt/A16793/2019 (H5N8)	98.7%
	EPI2162079	A/chicken/Kazakhstan/23/2020 (H5N8)	98%
	MW505413.1	A/Cygnus columbianus/Hubei/56/2020 (H5N8)	98.77%
	ON862694.1	A/duck/Shandong/SD0261/2021 (H5N6)	98.64%
	EPI1811626	A/chicken/Iraq/1/2020 (H5N8) segment 2 (PB1)	99%
PA	OP740951.1	A/Aviafauna/Kazakhstan/PA/2020 (H5N8)	99%
	EPI1881024	A/bean goose/Hubei/BQ11/2020 (A/H5N8) segment 3 (PA)	99%
	EPI2162080	A/chicken/Kazakhstan/23/2020 (A/H5N8) segment 3 (PA)	99%
HA	MW137842.1	A/Aviafauna/Kazakhstan/HA/2020 (H5N8)	98.10%
	OP740959.1	A/chicken/Egypt/F17229A/2019 (H5N8)	98.30%
	OQ711838.1	A/Migratory bird/India/01FE975/2021 (H5N8)	98.10%
	EPI1942903	A/Anser_Brachyrhynchus_Anser_Anser/Belgium/13846/2020 (A/H5N8) segment 4 (HA)	98%
NP	MT261706.1	A/duck/Egypt/A16793/2019 (H5N8)	99.27%
	OL354983.1	A/duck/Egypt/A19643/2021 (H5N8)	98.93%
	ON943055.1	A/chicken/Kazakhstan/23/2020 (H5N8)	98.80%
	EPI1811629	A/chicken/Iraq/1/2020(H5N8) segment 5 (NP)	98%
NA	MW137803.1	A/chicken/Egypt/F17230B/2019 (H5N8)	99.15%
	MZ882181.1	A/goose/China/21FU005/2020 (H5N8)	99.08%
	EPI1920391	A/white-tailed eagle/Germany-SH/AI02170/2020 (H5N8)	99%
	EPI1860070	A/anser_anser/Spain/297-1_21VIR1230-5/2021 (H5N8)	99%
	EPI1919630	A/barnacle goose/Germany-SH/AI02190/2020 (H5N8)	97%
MP	OP597600.1	Shelduck/Kalmykia/1814-1/2021 (H5N5)	98.78%
	OL353691.1	A/chicken/Egypt/A19670/2021 (H5N8)	98.78%
	OP597560.1	A/Dalmatian pelican/Astrakhan/417-1/2021 (H5N5)	98.67%
	EPI1920856	A/domestic goose/Germany-MV/AI02558/2021 (H5N8)	99%
	EPI1848412	A/common buzzard/Sweden/SVA210212SZ0284/KN000390/2021	99%
NS	MW505387.1	A/Cygnus columbianus/Hubei/51/2020 (H5N8)	98.21%
	EPI1913525	A/chicken/Egypt/AF12/2020 (H5N8) segment 8 (NS)	98%
	EPI1927723	A/pigeon/Kazakhstan/15-20-B-Talg-5/2020 (H5N8)	99%
	EPI2152264	A/brown-headed gull/Tibet/N38/2021 (H5N8) segment 8 (NS)	98%
	OR048707.1	A/mallard duck/Japan/KU-d89/2021 (H5N8)	98.09%
	MW137800.1	A/chicken/Egypt/F17230B/2019 (H5N8)	98.21%

**Table 4:** Evolutionary ancestors and subgroups of HPAI H5N8 viruses of the present study.

Gene	Evolutionary Ancestor	Subgroup	Subtype
HA	Egypt G6	Egypt G6	HPAI
	Local Endemic EuroII-2020 subclade Egy/ostrich/2021	EuroII-2020	
	Egypt G6 A/duck/Egypt/F91/2021	Egypt G6-like	
NA	Egypt G6 PA/chicken/Egypt/Elmonufia-backyard-AH/2019	Egypt G6-like	HPAI
	Egypt G6 A/chicken/Egypt/Alex-Breeder-AH/2021	Egypt G6-like	
	Local Endemic EuroII-2020 Egy/ostrich/2021	EuroII-2020	
M	A/chicken/Egypt/AF12/2020 (H5N8)	Russian-like H5N8 reassortant 2016	
	A/chicken/Kazakhstan/23/2020	EuroII-2020	HPAI
	A/shelduck/Kalmykia/18141/2021 (H5N5) A/Dalmatian pelican/Astrakhan/417-1/2021 (H5N5) A/pelican/Dagestan/397-1/2021 (H5N5	Russian reassortant H5N5 2021	
NP	A/chicken/Egypt/AF12/2020 (H5N8) A/chicken/Egypt/AF14/2020 (H5N8)	European-like H5N8 reassortant 2016	LPAI
	A/chicken/Egypt/A19670/2021	European-like H5N8 reassortant 2020	
PA	A/chicken/Kazakhstan/23/2020 A/chicken/Iraq/1/2020	Iraqi-like virus	HPAI
PB2	Eurasian LPAI A/duck/Shandong/SD0261/2021 (H5N6) A/chicken/China/2042/2020 (H9N2) Egy/ostrich/2021 H5N8	H9-like	LPAI
PB1	Eurasian LPAI A/duck/Shandong/SD0261/2021 (H5N6)	H9-like	LPAI
NS	A/chicken/Egypt/A19670/2021	Russian-like H5N8 reassortant 2016 and Russian-like H5N8 reassortant 2020 (Russian-like H5N8 reassortant 2016-like)	HPAI

## Data Availability

Data are available upon reasonable request from the corresponding authors.

## Supplementary Materials

The following supporting information can be downloaded at: https://www.mdpi.com/article/10.3390/v16111655/s1, Table S1. AIVs derived from ducks in Egypt between 2020/2021 and 2021/2022; Table S2. Summary of the genotyping of Egyptian AIV H5N8 genes analyzed in the current study with their accession numbers in GenBank; Table S3. Amino acid substitutions in the hemadsorbing sites and stalk region; Table S4. Comparison between Glycosylation sites of Neuraminidase (NA) of Egyptian viruses, the Egypt- ostrich-2021 and subclades of2.3.4.4 H5N8 b; Table S5. Amino acid differences between the three-reassortant H5N8 viruses and current study viruses; Table S6. Analysis of virulence determinants in the viral PB2, PB1, PA, NP, M2, NS1, and NS2 proteins in comparison with A/chicken/Egypt/NZ/2022 [65,90,91,92,93,94,95,96,97,98,99,100,101]. Table S7. Analysis of host range genetic determinants in the PB2, PB1, PA, NP, M1, M2, NS1, and NS2 proteins in H5N8 viruses in comparison with A/chicken/Egypt/NZ/2022 [58,88,90,92,93,94,102,103,104,105,106,107,108,109,110,111,112,113,114,115].
