# Supplementary material for: Phylogenetic Characterization of Novel Reassortant 2.3.4.4b H5N8 Highly Pathogenic Avian Influenza Viruses Isolated from Domestic Ducks in Egypt During the Winter Season 2021–2022
Source: Viruses. 2024 Oct 23;16(11):1655. doi: 10.3390/v16111655 (PMC11599000; doi:10.3390/v16111655)
Supplement: Supplementary file 1 [file viruses-16-01655-s001.zip › viruses-3185457-supplementary.pdf]

Supplementary Materials

Table S1. AIVs derived from ducks in Egypt between 2020/2021 and 2021/2022

Isolates	Breed	Age	Vaccine	Date of collection	Governorate
A/duck/Egypt/F653/10/2021	Muscovy	Two months			Dakahlia
A/duck/Egypt/F653/9/2021	Muscovy	one year			Damietta Governorate
A/duck/Egypt/F653/12/2021	Muscovy and Pekin	Five months			Gharbia
A/duck/Egypt/F653/13/2021	Muscovy and Pekin	One month	Not vaccinated		Gharbia
A/duck/Egypt/F653/18/2021	Pekin Duck	25 day old			Damietta
A/duck/Egypt/F653/19/2021	Native	55 day old			Damietta
A/duck/Egypt/F653/20/2021	Native	Six months			Damietta
A/duck/Egypt/F653/21/2021	Pekin duck	Eight months		2021 DEC	Dakahlia
A/duck/Egypt/F653/22/2021	Muscovy duck	one year			Dakahlia
A/duck/Egypt/F653/23/2021	Muscovy duck	Two months	vaccinated		Dakahlia
A/duck/Egypt/F653/24/2021	Muscovy duck	Three months			Kafer el sheikh
A/duck/Egypt/F653/26/2021	Pekin	25 day old			Kafer el sheikh
A/duck/Egypt/F653/28/2021	Muscovy duck	Nine months			Beheira
A/duck/Egypt/F653/29/2021	Muscovy duck	Two months			Damietta
A/duck/Egypt/F653/30/2021	Muscovy duck	Two months	Not vaccinated		Damietta
A/duck/Egypt/F653/31/2021	Moulard	Six months			Damietta
A/duck/Egypt/F653/25/2021	Sudan duck	Four months			Damietta

Table S2. Summary of the genotyping of Egyptian AIV H5N8 genes analyzed in the current study with their accession numbers in GenBank

H5N8 isolate	Genotypes	PB2	PB1	PA	HA	NP	NA	M	NS
A/duck/Egypt/F653/9/2021	G1	PQ416797	PQ421400	PQ416046	PQ395269	PQ412829	PQ412304	PQ412551	PQ421454
A/duck/Egypt/F653/10/2021	G2	PQ416798	PQ421401	PQ416047	PQ395270	PQ412830	PQ412305	PQ412552	PQ421455
A/duck/Egypt/F653/12/2021	G3	PQ416799	PQ421402	PQ416048	PQ395271	PQ412831	PQ412306	PQ412553	PQ421456
A/duck/Egypt/F653/13/2021	G4	PQ416800	PQ421403	PQ416049	PQ395272	PQ412832	PQ412307	PQ412554	PQ421457
A/duck/Egypt/F653/18/2021	G5	PQ416801	PQ421404	PQ416050	PQ395273	PQ412833	PQ412308	PQ412555	PQ421458
A/duck/Egypt/F653/19/2021	G1	PQ416802	PQ421405	PQ416051	PQ395274	PQ412834	PQ412309	PQ412556	PQ421459
A/duck/Egypt/F653/20/2021	G1	PQ416803	PQ421406	PQ416052	PQ395275	PQ412835	PQ412310	PQ412557	PQ421460
A/duck/Egypt/F653/21/2021	G1	PQ416804	PQ421407	PQ416053	PQ395276	PQ412836	PQ412311	PQ412558	PQ421461
A/duck/Egypt/F653/22/2021	G1	PQ416805	PQ421408	PQ416054	PQ395277	PQ412837	PQ412312	PQ412559	PQ421462
A/duck/Egypt/F653/23/2021	G4	PQ416806	PQ421409	PQ416055	PQ395278	PQ412838	PQ412313	PQ412560	PQ421463

A/duck/Egypt/F653/24/2021	G1	PQ416807	PQ421410	PQ416056	PQ395279	PQ412839	PQ412314	PQ412561	PQ421464
A/duck/Egypt/F653/26/2021	G1	PQ416808	PQ421411	PQ416057	PQ395280	PQ412840	PQ412315	PQ412562	PQ421465
A/duck/Egypt/F653/28/2021	G1	PQ416809	PQ421412	PQ416058	PQ395281	PQ412841	PQ412316	PQ412563	PQ421466
A/duck/Egypt/F653/29/2021	G1	PQ416810	PQ421413	PQ416059	PQ395282	PQ412842	PQ412317	PQ412564	PQ421467
A/duck/Egypt/F653/30/2021	G1	PQ416811	PQ421414	PQ416060	PQ395283	PQ412843	PQ412318	PQ412565	PQ421468
A/duck/Egypt/F653/31/2021	G1	PQ416812	PQ421415	PQ416061	PQ395284	PQ412844	PQ412319	PQ412566	PQ421469
A/duck/Egypt/F653/25/2021	G5	PQ416813	PQ421416	PQ416062	PQ395285	PQ412845	PQ412320	PQ412567	PQ421470

Table S3. Amino acid substitutions in the hemadsorbing sites and stalk region

	viruses	Deletion In Stalk Region	HB-Site																
			364-372									399-404					431-433		
			T	I	S	R	T	S	R	S	D	N	L	N	W	SG	P	E	E
**1.**	**Clade 2.3.4.4 B**	NO	T	I	S	R	T	S	R	S	D	N	L	N	W	SG	P	R	E
2.	EuroII2020	NO	T	I	S	R	T	S	R	S	D	N	L	N	W	SG	P	E	E
3.	Egy-ost-2021	NO	T	I	S	R	T	S	R	S	D	N	L	N	W	SG	P	E	E
4.	F653/9/2021	NO	T	I	S	R	T	S	R	S	D	N	L	N	W	SG	P	E	E
5.	F653/10/2021	NO	T	I	S	R	T	S	R	S	D	D	L	N	W	SG	P	E	E
6.	F653/12/2021	NO	T	I	S	R	T	S	R	S	D	D	L	N	W	SG	P	E	E
7.	F653/13/2021	NO	T	I	S	R	T	S	R	S	D	D	L	N	W	SG	P	E	E
8.	F653/18/2021	NO	T	I	S	R	N	S	R	S	D	N	L	N	W	SG	P	E	E
9.	F653/19/2021	NO	T	I	S	R	T	S	R	S	D	N	L	N	W	SG	P	E	E
10.	F653/20/2021	NO	T	I	S	R	T	S	R	S	D	N	L	N	W	SG	P	E	E
11.	F653/21/2021	NO	T	I	S	R	T	S	R	S	D	N	L	N	W	SG	P	E	E
12.	F653/22/2021	NO	T	I	S	R	T	S	R	S	D	N	L	N	W	SG	P	E	E
13.	F653/23/2021	NO	T	I	S	R	T	S	R	S	D	D	L	N	W	SG	P	E	E
14.	F653/24/2021	NO	T	I	S	R	T	S	R	S	D	D	L	N	W	SG	P	E	E
15.	F653/26/2021	NO	T	I	S	R	T	S	R	S	D	N	L	N	W	SG	P	E	E
16.	F653/28/2021	NO	T	I	S	R	T	S	R	S	D	N	L	N	W	SG	P	E	E
17.	F653/29/2021	NO	T	I	S	R	T	S	R	S	D	N	L	N	W	SG	P	E	E
18.	F653/30/2021	NO	T	I	S	R	T	S	R	S	D	N	L	N	W	SG	P	E	E
19.	F653/31/2021	NO	T	I	S	R	T	S	R	S	D	N	L	N	W	SG	P	E	E
20.	F653/25/2021	NO	T	I	S	R	N	S	R	S	D	N	L	N	W	SG	P	E	E

Table S4. Comparison between Glycosylation sites of Neuraminidase (NA) of Egyptian viruses, the Egypt- ostrich-2021 and subclades of2.3.4.4 H5N8 b

	Glycosylation sites								
	**2**	**46**	**54**	**54**	**67**	**84**	**144**	**293**	**398**
2.3.4.4a		NGTV	NETV		NTSV	NNTE	NGTV	NWTG	NWSG
2.3.4.4b			NETV		NTSV	NGTV	NGTV	NW TG	NWSG
EuroII2020			NETV		NTSV	NGTV	NGTV		NWSG
Egy-ost-2021			NETV		NTSV	NGTV	NGTV		NWSG
/F653/13			NETV	NETA	NTSV	NGTV	NGTV		NWSG
/F653/29	NPSQ		NETV		NTSV	NGTV	NGTV		NWSG

Table S5. Amino acid differences between the three-reassortant H5N8 viruses and current study viruses

Protein	A	A/common-coot/Egypt/CA285/2016	A/duck/Egypt/SS19/2017	A/chicken/Egypt/NZ/2022	Current study
HA	54	D	D	N	D
	140	T	T	A	A
	175	L	M	L	L
	236	N	N	D	(11/17) D<br>(6/17) N
	268	G	E	G	G
	269	V	V	M	V
	306	V	V	L	V
	522	V	V	A	A
NA	106	V	V	I	I
	191	I	V	I	(16/17) I<br>(1/17) V
	201	V	V	I	I
	213	V	V	I	(2/17) I<br>(15/17) V
	214	V	F	V	V
	245	A	A	S	S
	265	T	T	A	A
	295	T	T	M	M
	342	Q	L	Q	Q (16/17)<br>H (1/17)
	359	V	V	M	M
PB2	70	L	R	R	R
	71	K	N	N	N
	255	I	V	V	V
	288	H	Q	Q	Q
	292	I	V	V	I (16/17)<br>V (1/17)
	344	V	I	I	I
	354	I	I	V	V
	451	V	I	I	I
	464	F	L	L	L
	466	N	D	D	D
	615	I	I	V	I
	678	D	D	Y	Y
	714	G	S	G	S

Table S5. Cont.

Protein	A	A/common-coot/Egypt/CA285/2016	A/duck/Egypt/SS19/2017	A/chicken/Egypt/NZ/2022	Current study
PB1	110	A	T	A	A
	113	V	V	I	I
	152	S	L	S	S
	168	R	K	R	R
	211	R	R	K	R
	374	T	A	T	T
	405	L	P	P	P
	407	V	M	M	M
	431	H	Y	Y	Y (16/17)<br>H (1/17)
	464	N	D	D	D
	473	L	V	V	V
	576	I	L	L	L
	584	H	R	R	R
	591	V	V	I	I
	628	L	M	L	L
	633	N	S	S	S
	636	D	E	E	E
	645	I	V	V	V

	654	N	S	S	S
	691	K	K	R	K
	694	N	S	S	S
PB1-F1	42	Y	C	Y	W
	46	M	T	M	S
	48	Q	R	Q	Q

Table S5. Cont.

Protein	A	A/common-coot/Egypt/CA285/2016	A/duck/Egypt/SS19/2017	A/chicken/Egypt/NZ/2022	Current study
PA	27	D	N	D	D
	57	Q	R	R	R
	59	G	G	E	E
	96	N	N	H	N
	101	E	D	D	E
	113	K	K	R	R
	120	I	I	V	I
	183	A	V	A	A
	208	T	K	T	T
	211	M	L	M	M
	214	L	L	F	L
	308	I	I	T	T
	348	I	I	F	I
	367	R	K	K	K
	432	V	I	V	V
	445	V	I	I	Y
	665	M	L	L	L
NP	214	K	R	R	R
	312	V	I	V	V
	318	P	S	P	P 17l17
M1	33	V	A	A	A
	42	L	L	I	L
	85	S	N	N	N
	134	R	R	K	R
	139	A	T	T	T
	209	K	Q	Q	A
	248	M	L	M	L
M2	18	K	N	R	N
	95	E	E	E	E
	96	L	L	L	L

Table S5. Cont.

Protein	A	A/common-coot/Egypt/CA285/2016	A/duck/Egypt/SS19/2017	A/chicken/Egypt/NZ/2022	Current study
NS1	82	A	D	A	A
	100	R	R	K	R
	165	S	F	S	S
	205	N	S	N	N
	210	D	N	D	G
	214	F	L	L	L
	215	S	P	P	(5/17) P (12/17) S
NS2	48	T	A	T	V
	82	E	G	E	E

A= Alanine, R= Arginine, N= Asparagine, D= Aspartic acid, C= Cysteine, Q= Glutamine, E= Glutamic acid, G= Glycine, H= Histidine, I= Isoleucine, K= Lysine, M= Methionine, F= Phenylalanine, P= Proline, S= Serine, T= Threonine, W= Tryptophan, Y= Tyrosine, and V= Valine.

Table S6. Analysis of virulence determinants in the viral PB2, PB1, PA, NP, M2, NS1, and NS2 proteins in comparison with A/chicken/Egypt/NZ/2022

Protein	A.A site	Virulent	Avirulent	Current study	ON847347-A/chicken/Egypt/NZ/2022	Ref.
PB2	627	K	E	E (17/17)	E	[90]
	147	L	M	L (17/17)	I	[90]
	250	G	V	V (17/17)	V	[90]
	504	V	I	V (17/17)	V	[91]
	701	N	D	D (17/17)	D	[92]
	591	K	Q	Q (17/17)	Q	[93]
PB1	317	I	M/V	I (13/17) M (4)	M	[94,95]
PA	127	V	I	V (17/17)	V	[96]
	672	L	F	L (17/17)	L	[97]
	100	R	V	V (17/17)	V	[98]
	550	L	I	L (17/17)	L	[91]
NP	470	R	K	K (17/17)	K	[99]
M2	64	S/A/F	p	F (17/17)	S	[96]
	69	P	L	P (17/17)	P	[96]
NS1	42	S	A/P	S (17/17)	S	[64]
	92	E	D	D (17/17)	D	[95]
	103	L	F	F (17/17)	F	[100]
	106	I	M	M (17/17)	M	[100]
	189	N	D/G	N (17/17)	D	[101]
PDZ motif (227–230)		Presence	Deletion	Deletion		[101]
NS2	31	I	M	I (17/17)	M	[101]
	56	Y	H/L	H (17/17)	H	[101]

A= Alanine, R= Arginine, N= Asparagine, D= Aspartic acid, C= Cysteine, Q= Glutamine, E= Glutamic acid, G= Glycine, H= Histidine, I= Isoleucine, K= Lysine, M= Methionine, F= Phenylalanine, P= Proline, S= Serine, T= Threonine, W= Tryptophan, Y= Tyrosine, and V= Valine.

Table S7. Analysis of host range genetic determinants in the PB2, PB1, PA, NP, M1, M2, NS1, and NS2 proteins in H5N8 viruses in comparsion with A/chicken/Egypt/NZ/2022

Protein	A.A site	Avian preference	Mammalian preference	Current study	ON847347-A/chicken/Egypt/NZ/2022	Ref.
PB2	44	A	S	A (17/17)	A	[94,102]
	64	M	T	M (17/17)	M	[103]
	81	T	M	T (17/17)	T	[102]
	199	A	S	A (17/17)	A	[94,102]
	591	Q	K	Q (17/17)	Q	[93]
	627	E	K	E (17/17)	E	[90]
	661	A	T	A (17/17)	A	[104]
	667	V	I	A (17/17)	V	[105,106]
	701	D	N	D (17/17)	D	[92]
	702	K	R	K (17/17)	K	[104]
PB1	13	L	P	P (17/17)	P	[105]
	336	V	I	V (17/17)	V	[107]
	375	N	S	N (17/17)	N	[107]
	598	L	P	L (17/17)		[88]
PA	28	P	L	P (17/17)	P	[108]
	55	D	N	D (17/17)	D	[94,102]
	57	R	Q	R (17/17)	R	[105]
	100	V	A	V (17/17)	V	[109]
	133	E	G	E (17/17)	G	[110]
	225	S	C	S (17/17)	C	[58]
	241	C	Y	C (17/17)	C	[111]
	268	L	I	L (17/17)	L	[58]
	356	K	R	K (17/17)	K	[105]
	382	E	D	E (17/17)	E	[58]
	404	A	S	A (17/17)	A	[105]
	409	S	N	S (17/17)	S	[94,102]
	552	T	S	T (17/17)	T	[58]
	615	K	L	R (17/17)	R	[111]

Table S7. Cont.

Protein	A.A site	Avian preference	Mammalian preference	Current study	ON847347-A/chicken/Egypt/NZ/2022	Ref.
NP	33	V	I	V (17/17)	V	[98]
	16	G	D	G (17/17)	G	[112]
	61	I	L	I (17/17)	I	[58]
	109	I	V	I (17/17)	I	[94]
	136	L	M	L (17/17)	L	[102]
	214	R	K	R (7/17)	R	[94,102]
	313	F	Y	F (17/17)	F	[94,102]
	357	Q	K	Q (17/17)	Q	[94]
	372	E	D	E (17/17)	E	[94]
	398	K	Q	Q (17/17)	Q	[94]
	455	D	E	D (17/17)	D	[94]
M1	15	V	I	V(17/17)	V	[113]
	115	V	I	V (17/17)	V	[58]
	121	T	A	T (17/17)	T	[58,113]
	137	T	A	A 2/17 T 15/17	T	[102]

M2	11	T	I	T (17/17)	T	[94]
	16	E	G/D	E (17/17)	E	[58,102]
	20	S	L	L (17/17)	S	[94,102]
	28	I	I/V	I (17/17)	I	[102]
	57	Y	H	K (17/17)	Y	[94]
	55	L	F	L (17/17)	L	[114]
	86	V	A	A 1/17 V /17	V	[105]
NS1	227	E	K/R	Deleted		[115]
	full length	217	230	217		[115]
NEP	70	S	G	S (2/17) G (15/17)	G	[105,106]

A= Alanine, R= Arginine, N= Asparagine, D= Aspartic acid, C= Cysteine, Q= Glutamine, E= Glutamic acid, G= Glycine, H= Histidine, I= Isoleucine, K= Lysine, M= Methionine, F= Phenylalanine, P= Proline, S= Serine, T= Threonine, W= Tryptophan, Y= Tyrosine, and V= Valine.

Table S6. Analysis of virulence determinants in the viral PB2, PB1, PA, NP, M2, NS1, and NS2 proteins in comparison with A/chicken/Egypt/NZ/2022

Protein	A.A site	Virulent	Avirulent	Current study	ON847347-A/chicken/Egypt/NZ/2022	Ref.
PB2	627	K	E	E (17/17)	E	[90]
	147	L	M	L (17/17)	I	[90]
	250	G	V	V (17/17)	V	[90]
	504	V	I	V (17/17)	V	[91]
	701	N	D	D (17/17)	D	[92]
	591	K	Q	Q (17/17)	Q	[93]
PB1	317	I	M/V	I (13/17) M (4)	M	[94,95]
PA	127	V	I	V (17/17)	V	[96]
	672	L	F	L (17/17)	L	[97]
	100	R	V	V (17/17)	V	[98]
	550	L	I	L (17/17)	L	[91]
NP	470	R	K	K (17/17)	K	[99]
M2	64	S/A/F	p	F (17/17)	S	[96]
	69	P	L	P (17/17)	P	[96]
NS1	42	S	A/P	S (17/17)	S	[65]
	92	E	D	D (17/17)	D	[95]
	103	L	F	F (17/17)	F	[100]
	106	I	M	M (17/17)	M	[100]
	189	N	D/G	N (17/17)	D	[101]
PDZ motif (227–230)		Presence	Deletion	Deletion		[101]
NS2	31	I	M	I (17/17)	M	[101]
	56	Y	H/L	H (17/17)	H	[101]

A= Alanine, R= Arginine, N= Asparagine, D= Aspartic acid, C= Cysteine, Q= Glutamine, E= Glutamic acid, G= Glycine, H= Histidine, I= Isoleucine, K= Lysine, M= Methionine, F= Phenylalanine, P= Proline, S= Serine, T= Threonine, W= Tryptophan, Y= Tyrosine, and V= Valine.

Table S7. Analysis of host range genetic determinants in the PB2, PB1, PA, NP, M1, M2, NS1, and NS2 proteins in H5N8 viruses in comparsion with A/chicken/Egypt/NZ/2022

Protein	A.A site	Avian preference	Mammalian preference	Current study	ON847347-A/chicken/Egypt/NZ/2022	Ref.
PB2	44	A	S	A (17/17)	A	[94,102]
	64	M	T	M (17/17)	M	[103]
	81	T	M	T (17/17)	T	[102]
	199	A	S	A (17/17)	A	[94,102]
	591	Q	K	Q (17/17)	Q	[93]
	627	E	K	E (17/17)	E	[90]
	661	A	T	A (17/17)	A	[104]
	667	V	I	A (17/17)	V	[105,106]
	701	D	N	D (17/17)	D	[92]
	702	K	R	K (17/17)	K	[104]
PB1	13	L	P	P (17/17)	P	[105]
	336	V	I	V (17/17)	V	[107]
	375	N	S	N (17/17)	N	[107]
	598	L	P	L (17/17)		[88]
PA	28	P	L	P (17/17)	P	[108]
	55	D	N	D (17/17)	D	[94,102]
	57	R	Q	R (17/17)	R	[105]
	100	V	A	V (17/17)	V	[109]
	133	E	G	E (17/17)	G	[110]
	225	S	C	S (17/17)	C	[58]
	241	C	Y	C (17/17)	C	[111]
	268	L	I	L (17/17)	L	[58]
	356	K	R	K (17/17)	K	[105]
	382	E	D	E (17/17)	E	[58]
	404	A	S	A (17/17)	A	[105]
	409	S	N	S (17/17)	S	[94,102]
	552	T	S	T (17/17)	T	[58]
	615	K	L	R (17/17)	R	[111]

Table S7. Cont.

Protein	A.A site	Avian preference	Mammalian preference	Current study	ON847347-A/chicken/Egypt/NZ/2022	Ref.
NP	33	V	I	V (17/17)	V	[98]
	16	G	D	G (17/17)	G	[112]
	61	I	L	I (17/17)	I	[58]
	109	I	V	I (17/17)	I	[94]
	136	L	M	L (17/17)	L	[102]
	214	R	K	R (7/17)	R	[94,102]
	313	F	Y	F (17/17)	F	[94,102]
	357	Q	K	Q (17/17)	Q	[94]
	372	E	D	E (17/17)	E	[94]
	398	K	Q	Q (17/17)	Q	[94]
	455	D	E	D (17/17)	D	[94]
M1	15	V	I	V(17/17)	V	[113]
	115	V	I	V (17/17)	V	[58]
	121	T	A	T (17/17)	T	[58,113]
	137	T	A	A 2/17 T 15/17	T	[102]

M2	11	T	I	T (17/17)	T	[94]
	16	E	G/D	E (17/17)	E	[58,102]
	20	S	L	L (17/17)	S	[94,102]
	28	I	I/V	I (17/17)	I	[102]
	57	Y	H	K (17/17)	Y	[94]
	55	L	F	L (17/17)	L	[114]
	86	V	A	A 1/17 V /17	V	[105]
NS1	227	E	K/R	Deleted		[115]
	full length	217	230	217		[115]
NEP	70	S	G	S (2/17) G (15/17)	G	[105,106]

A= Alanine, R= Arginine, N= Asparagine, D= Aspartic acid, C= Cysteine, Q= Glutamine, E= Glutamic acid, G= Glycine, H= Histidine, I= Isoleucine, K= Lysine, M= Methionine, F= Phenylalanine, P= Proline, S= Serine, T= Threonine, W= Tryptophan, Y= Tyrosine, and V= Valine.
